# Localization *in vivo and in vitro* confirms EnApiAP2 protein encoded by *ENH_00027130* as a nuclear protein in *Eimeria necatrix*

**DOI:** 10.3389/fcimb.2023.1305727

**Published:** 2023-12-05

**Authors:** Weimin Cai, Qianqian Feng, Liyue Wang, Shijie Su, Zhaofeng Hou, Dandan Liu, Xilong Kang, Jinjun Xu, Zhiming Pan, Jianping Tao

**Affiliations:** ^1^ College of Veterinary Medicine, Yangzhou University, Yangzhou, China; ^2^ Jiangsu Co-innovation Center for Prevention and Control of Important Animal Infectious Diseases and Zoonoses, Yangzhou University, Yangzhou, China; ^3^ Jiangsu Key Laboratory of Zoonosis, Yangzhou University, Yangzhou, China; ^4^ Principal's Office, Suqian University, Suqian, China

**Keywords:** *Eimeria*, EnApiAP2, localization, NLS, mAb

## Abstract

**Introduction:**

Apicomplexan AP2 family of proteins (ApiAP2) are transcription factors (TFs) that regulate parasite growth and development, but little is known about the ApiAP2 TFs in *Eimeria* spp. *ENH_00027130* sequence is predicted to encode a *Eimeria necatrix* ApiAP2 protein (EnApiAP2).

**Methods:**

The cDNAs encoding full-length and truncated EnApiAP2 protein were cloned and sequenced, respectively. Then, the two cDNAs were cloned into the pET28a(+) expression vector and expressed expressed in *Escherichia coli* BL21. The mouse polyclonal antibody (pAb) and monoclonal antibody (mAb) against recombinant EnApiAP2 (rEnApiAP2) and EnApiAP2tr (rEnApiAP2tr) were prepared and used to localize the native EnApiAP2 protein in *E. necatrix*, respectively. Finally, the recombinant pEGFP-C1-ΔNLS-EnApiAP2s (knockout of a nuclear localization sequence, NLS) and pEGFP-C1-EnApiAP2 plasmid were constructed and transfected into DF-1 cells, respectively, to further observe subcellular localization of EnApiAP2 protein.

**Results:**

The En*ApiAP2* gene had a size of 5019 bp and encoded 1672 amino acids, containing a conserved AP2 domain with a secondary structure consisting of an α-helix and three antiparallel β-strands. The rEnApiAP2 and rEnApiAP2tr were predominantly expressed in the form of inclusion bodies, and could be recognized by the 6×His tag mAb and the serum of convalescent chickens after infection with *E. necatrix*, respectively. The native EnApiAP2 protein was detected in sporozoites (SZ) and second generation merozoites (MZ-2) extracts, with a size of approximately 210 kDa. A quantitative real-time PCR (qPCR) analysis showed that the transcription level of En*ApiAP2* was significantly higher in SZ than in MZ-2, third generation merozoites (MZ-3) and gametocytes (*P*<0.01). EnApiAP2 protein was localized in the nuclei of SZ, MZ-2 and MZ-3 of *E. necatrix*. The protein of EnApiAP2 was localized in the nucleus of the DF-1 cells, whereas the ΔNLS-EnApiAP2 was expressed in the cytoplasm, which further confirmed that EnApiAP2 is nucleoprotein.

**Discussion:**

EnApiAP2 protein encoded by *ENH_00027130* sequence was localized in the nucleus of *E. necatrix* parasites, and relied on the NLS for migration to DF-1 cell nucleus. The function of EnApiAP2 need further study.

## Introduction

*Eimeria* spp. are protozoan parasites of the phylum Apicomplexa responsible for coccidiosis, a ubiquitous intestinal disease of livestock that has major impacts on animal welfare and agroeconomics (Arisue and Hashimoto, 2015). For example, coccidiosis has caused substantial losses to the chicken industry, including losses in the production process as well as prevention and treatment costs, which are calculated to be around £10.4 billion globally at 2016 prices, equivalent to £0.16 per chicken produced (Blake et al., 2020). At present, good husbandry practices, anticoccidial drugs and/or live anticoccidial vaccines are used to control coccidiosis. (Saeed and Alkheraije, 2023). However, the extensive use of drugs has inevitably led to the emergence of drug resistance and drug residues in the food chain and the environment. Non-attenuated vaccines carry the potential risk of vaccine-induced disease. The high costs of attenuated vaccines limit their widespread use (Gao et al., 2023). It is therefore essential to continue to research and develop innovative strategies to effectively control coccidiosis in poultry.

Like other apicomplexan parasites, *Eimeria* spp. have a complex life cycle involving merogony (also known as schizogony) and gametogony in the host, and sporogony (or sporulation) *in vitro* (Burrell et al., 2020). The developmental cycle begins with the ingestion of sporulated oocysts by host. If ingested by hosts such as a chicken, the sporulated oocyst will release sporocysts. Subsequently the sporocysts enter the small intestine, where enzymatic digestion releases the sporozoites. The sporozoites begin two to three rounds of asexual replication, known as schizogony, to produce merozoites by migrating to their preferred site of development to initiate cellular invasion. MZ-2 or MZ-3 then form the GAM, which mediate fertilization and produce the next generation of oocysts for excretion in the faeces. The oocysts mature into infective sporulated oocyst in the external environment (Walker et al., 2013; Burrell et al., 2020; Lopez-Osorio et al., 2020). Clearly, for the survival, reproduction and transmission of parasites within the Apicomplexa phylum, the process of differentiation from one stage to the next is critical. However, the mechanisms involved in the transformation from one developmental stage to the next are not fully understood.

Apicomplexan AP2 family of protein (ApiAP2) is a single validated class of apicomplexan TFs, initially identified in the genera *Cryptosporidium*, *Plasmodium* and *Theileria* (Balaji et al., 2005), and have been subsequently identified in all apicomplexan genomes analyzed to date (Kim, 2018; Jeninga et al., 2019; Chen et al., 2023). In *Eimeria*, the number of genes containing AP2 domains was found to vary from 44 to 54 (Reid et al., 2014). ApiAP2 modulate key regulatory decisions at all stages of parasite development (Balaji et al., 2005; Campbell et al., 2010), and play an essential role in the conversion of *Plasmodium* (Jofuku et al., 1994),*Cryptosporidium parvum* (Oberstaller et al., 2014), *Toxoplasma gondii* (White et al., 2014) and *Theileria annulata* (Pieszko et al., 2015) from asexual to sexual replication. However, a recent study showed that *ETH2_0411800*, a sporogonic stage-specific *ApiAP2* gene in *E. tenella*, is not essential for the growth and development of *E. tenella*, as the overexpression and knockout strains showed no significant differences in oocyst size or production compared to the parental strain (Chen et al., 2023). Therefore, the ApiAP2 functions of eimerian parasites require further study.

*Eimeria necatrix* is a highly pathogenic coccidian that can cause high mortality in susceptible birds, particularly in chickens over 8 weeks old reared on litter floors (Mesa-Pineda et al., 2021). In our previous studies on the comparative transcriptome analysis of different developmental stages of *E. necatrix* (Su et al., 2017; Su et al., 2018; Gao et al., 2021a), we detected 37 transcripts contained AP2 domains, of which 2, 5, 7 and 12 transcripts were upregulated in SZ, MZ-2, MZ-3 and GAM, respectively. The expression of AP2 family TFs throughout the life cycle suggests that members of this family are crucial regulators of gene expression at all stages of *E. necatrix* development.

In the present study, an *ApiAP2* gene (*ENH_00027130*) was cloned and expressed in *Escherichia coli* BL21 (DE3) using pET28a(+), as an expression vector. The native EnApiAP2 protein and its subcellular localization in *E. necatrix* was analyzed by Western blot and indirect immunofluorescence assay (IFA), respectively. The nuclear localization characteristics of EnApiAP2 was further confirmed in DF-1 cells. Our findings provide a basis for understanding of the role of EnApiAP2 protein regulating *E. necatrix* development.

## Materials and methods

### Parasites and animals

The Yangzhou *E. necatrix* strain used in this study was originally isolated from *E. necatrix*-infected chickens in 2009 (Yangzhou, Jiangsu, China). The identity of the strain was determined by the single oocyst method and confirmed by microscopic examination and sequence analysis of the internal transcribed spacer region of the ribosomal RNA (rRNA) gene (Liu et al., 2014).

Yellow-feathered broilers were obtained from the Jiangsu Jinghai Poultry Industry Group Co., Limited (Nantong, Jiangsu, China). The chickens were housed in *Eimeria*-free isolation cages and were provided with clean water and adequate feed in the absence of anticoccidial drugs. Chicken faeces were collected and analyzed by salt flotation and light microscopy to confirm the absence of oocysts in each chicken prior to experimental inoculations. Chickens between 4 and 5 weeks of age were used to prepare SZ, MZ-2, MZ-3 and GAM of *E. necatrix*. Six-week-old female BALB/c mice free of specific pathogens were purchased from Yangzhou University (Centre for Comparative Medicine) and maintained under conditions free of specific pathogens.

All animal care and procedures were performed in accordance with the guidelines for the use of animals in toxicology. The Animal Care and Use Committee of the College of Veterinary Medicine, Yangzhou University approved the study protocol.

### Preparation of parasites

Oocysts were isolated from the feces of chickens infected with *E. necatrix* oocysts, and SZ were purified from sporulated oocysts using a DEAE-52 cellulose chromatographic column (Whatman, Kent, UK) according to the method described by Gao et al. (2021b). MZ-2 were isolated from the small intestine of chickens at 136 h post-infection (HPI), and purified by density gradient centrifugation according to the method described by Su et al. (2017). MZ-3 were isolated from the caca of chickens at 144 HPI, and purified according to the method described by Su et al. (2017). GAM were isolated from the cecal mucosal tissue of chickens infected with MZ-2 at 32 ± 0.5 HPI, and purified according to the method described by Su et al. (2018). The purified parasites were frozen immediately in liquid nitrogen for future use.

### Total RNA extraction

Total RNA was isolated from purified SZ, MZ-2, MZ-3 and GAM using FastPure^®^ Cell/Tissue Total RNA Isolation Kit (Vazyme, Nanjing, China) according to the manufacturer’s instructions, then resuspended in diethylpyrocarbonate-treated water and quantified using a UV spectrophotometer (NanoDrop2000c; Thermo, Waltham, USA) and stored at -80°C for further use.

### Cloning and bioinformatics analysis of full-length En*ApiAP2* gene

**Figure 1:**
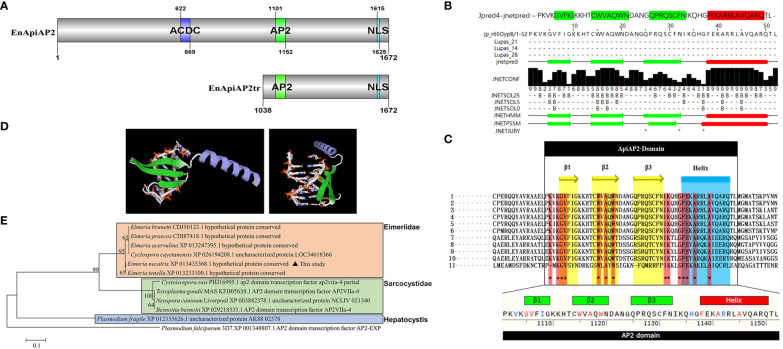
Sequence analysis of En*ApiAP2*. **(A)** Schematic diagram of the structural domain of EnApiAP2. **(B)** The secondary structure of AP2 domain region of EnApiAP2 predicted using Jpred4 software. **(C)** Comparison of the secondary structure of AP2 domain between *E*. *necatrix* (1, XP_013435368.1), *E. tenella* (2, XP_013233100.1), *E. praecox* (3, CDI87418.1), *E. acervulina* (4, XP_013247395.1), *E. brunetti* (5, CDJ50123.1), *Cyclospora cayetanensis* (6, XP_026194208.1), *Neospora caninum* (7, XP_003882378.1), *Toxoplasma gondii* (8, KFH05638.1), *Besnoitia besnoiti* (9, XP_029218535.1), *Cystoisospora suis* (10, PHJ16995.1) and *Plasmodium fragile* (11, CDI87418.1). **(D)** The tertiary structure of AP2 domain of EnApiAP2 predicted using Swiss-model software. **(E)** Phylogenetic tree of EnApiAp2 and homologous proteins, which were inferred from their conserved AP2 domain amino acid sequences using the neighbor-joining method (NJ). "*" represents 100% homology.

The sequence of the gene encoding the EnApiAP2 protein was amplified by reverse transcription polymerase chain reaction (RT-PCR) using the MZ-2 cDNA as a template and the PhantaTM Max Super-Fidelity DNA Polymerase instructions (Vazyme, Nanjing, China) according to the manufacturer’s instructions. Specific primer sequences (**Table 1**) were used at 10 pmol-μL-1 each to amplify the target gene under the following conditions: an initial denaturation step at 94°C for 3 min; followed by 35 cycles of 94°C for 15 s, 62°C for 15 s, 72°C for 2.5 min and a final extension step at 72°C for 10 min. The PCR products were analyzed by 1.0% agarose gel electrophoresis (**Figure 1**) and then purified and cloned into the pMD™18-T vector (Takara, Dalian, China) according to the manufacturer’s instructions. The recombinant plasmid pMD™18-T-EnApiAP2s were then transformed into chemically competent *E. coli* DH5α cells (Transgen, Beijing, China) (Green and Sambrook, 2017). Positive clones were selected for sequencing by a commercial company (BGI, Beijing, China).

**Table 1:** Sequences of primers.

Primer name	Sequence 5’-3’
EnApiAP2-T-F	ATGCGCGCGAATCGGTCCGTG
EnApiAP2-T-R	TCAAATGCTGGGTGCCTCG
EnApiAP2tr-T-F	ATGGCTGAAGGTTCTGGTGT
EnApiAP2tr-T-R	TCAAATGCTGGGTGCCCTCG
EnApiAP2-pET-F	agcaaatgggtcgcggatccgaattcATGCGCGAATCGGTCCGTG
EnApiAP2-pET-R	gtgctcgagtgcggccgcaagcttTCAAATGCTGGGTGCCCTCG
Linear-pET-F	AAGCTTGCGGCCGCACTCGAGCAC
Linear-pET-F	GAATTCGGATCCGCGACCCATTTGCT
EnApiAP2tr-pET-F	CCCGGATCCATGGCTGAAGGTTCTGGTGTT
EnApiAP2tr-pET-R	CCGGAATTCTCAAATGCTGGGTGCCCT
EnApiAP2-TYGFP-F	tacaagtccggactcagatctATGCGCGAATCGGTCCGTGC
EnApiAP2-TYGFP-ΔNLS-A2	tgaatttactaaaacttcttcTTCCGCTTCCAACCC
EnApiAP2-TYGFP-ΔNLS-B1	gggttggaagcggaagaaGAAGTTTTAGTAAATTCA
EnApiAP2-TYGFP-R	gtaccgtcgactgcagaattcTCAAATGCTGGGTGCCCTCGACA
Linear-GFP-F	GAATTCTGCAGTCGACGG
Linear-GFP-R	AGATCTGAGTCCGGA
EGFPC-F	AGCACCCAGTCCGCCCTGAGC
SV40pA-R	GAAATTTGTGATGCTATTGC
qEnApiAP2-F	CAACTCGGCAGGAAACGGAT
qEnApiAP2-R	TAGCTTCTCCCGGAGTGAAACTGA
qEn5.8S-F	TTCATACTGCGTCTAATGCACC
qEn5.8S-R	CGAGTCCCTACCGCAGTACTA

The sequencing results were analyzed with BLASTN, and the protein sequences were predicted with DNAStar software Lasergene 7.0. The conserved-domains were predicted with ToxoDB and NCBI webpage (https://www.ncbi.nlm.nih.gov/Structure/cdd/wrpsb.cgi). The secondary structure of the conserved domain of EnApiAP2 was predicted with Jpred (http://www.compbio.dundee.ac.uk/jpred4/cgi-bin/chklog?keywords=jp:BsNYvM) and the 3D structure of the conserved domain of EnApiAP2 was predicted with Swiss-model (https://swissmodel.expasy.org/interactive). The post translational modification sites (PTMs) of EnApiAP2 proteins were predicted with GPS 6.0 (http://gps.biocuckoo.cn/index.php). The signal peptide and transmembrane region of EnApiAP2 was predicted with SignalP-5.0 (http://www.cbs.dtu.dk/services/SignalP-5.0/) and TMHMM (http://www.cbs.dtu.dk/services/TMHMM/), respectively. The subcellular localization were predicted with Uniport (https://www.uniprot.org/uniprotkb/P06213/entry#subcellular_location) and Cell-PLoc-2 (http://www.csbio.sjtu.edu.cn/bioinf/Cell-PLoc-2/). The phylogenetic trees based on Neighbor Joining analysis were constructed with 1 000 bootstrap tests with MEGA5 (Cai et al., 2022), *Plasmodium falciparum* (PF14_0633) was selected as an outgroup. The nuclear localization sequence (NLS) of EnApiAP2 were predicted with PSORT (https://www.genscript.com/psort.html) and cNLS Mapper (https://nls-mapper.iab.keio.ac.jp/cgi-bin/NLS_Mapper_form.cgi).

### Cloning of truncated En*ApiAP2* gene

A 1908 nucleotide-long sequence (from 3112 bp to 5019 bp of En*ApiAP2* gene, aa residues 1038–1672) containing AP2 domain was amplified by RT-PCR with a pair of specific primers (**Table 1**) using the method as above. The products were purified and cloned into the pMD™18-T vector (Takara, Dalian, China) following the manufacturer’s instructions. The recombinant plasmid pMD18-T-EnApiAP2tr were then transformed into chemically competent DH5α *E coli* cells (Transgen, Beijing, China) (Green and Sambrook, 2017). Positive clones were selected for sequencing by BGI (BGI, Beijing, China).

### Expression and purification of rEnApiAP2 and rEnApiAP2tr

According to the sequencing results, specific primers (**Table 1**) containing homologous arms and restriction sites for *Eco*R I and *Hin*d III were designed to amplify the sequence encoding EnApiAP2 and EnApiAP2tr, respectively. The product of EnApiAP2 was inserted into pET28a(+) which was linearized by reverse primers (**Table 1**) as skeleton vector and the homologous recombination was performed according to ClonExpress^®^ II One Step Cloning kit (Vazyme, Nanjing, China). The product of EnApiAP2tr was inserted into pET28a(+) using T4 ligase. The recombinant plasmids pET28a(+)-EnApiAP2 and pET28a(+)-EnApiAP2tr were selected for kanamycin resistance. They were sequenced and used to transform *E. coli* BL21 cells. Recombinant EnApiAP2 and EnApiAP2tr (rEnApiAP2 and rEnApiAP2tr) were respectively expressed and purified as described previously (Liu et al., 2014). Briefly, the expression strain pET28a(+)-EnApiAP2 and pET28a(+)-EnApiAP2tr were induced for 4 h at a final concentration of 1.0 mM IPTG and 0.6 mM IPTG, respectively. The induced bacteria were subjected to ultrasonic treatment in a lysis buffer (ON 2 s/OFF 3 s, power 30%, lasting for 5 min). Recombinant proteins were collected through a Ni-NTA affinity chromatography column (Genscript, Nanjing, China). Purified protein samples were separated by 6% and 12% SDS-PAGE and stained with Coomassie blue. The yields of purified proteins were estimated using a NanoDrop2000c (Thermo, Waltham, U.S.A).

### Preparation of pAb against rEnApiAP2 and rEnApiAP2tr

Mouse anti-rEnApiAP2 and rEnApiAP2tr polyclonal antibodies (pAbs) were prepared as previously described (Liu et al., 2014). Briefly, the 50 μg proteins, resuspended in 50 μL PBS and mixed with 50 μL Quick Antibody-Mouse 3W (Biodragon, Beijing, China), were used to immunize 6-week-old BALB/c mice twice according to the manufacturer’s recommendations. The sterile PBS mixed with QuickAntibody-Mouse3W was used as control. Blood was collected 7 days after the second immunization. The mouse pAb was isolated by centrifugation at 1500 × g for 15 min and stored at -80°C.

As described in a previous study (Liu et al., 2014), antibody levels were determined by enzyme-linked immunosorbent assay (ELISA) (Liu et al., 2014). Briefly, recombinant proteins were coated onto 96-well microtiter plates (1 μg/well) and incubated overnight at 4°C. After blocking with 1% BSA for 1 h, the plates were incubated with PBS-diluted mouse sera for 1 h at 37°C. After three 5-minute washes with PBST, HRP-conjugated goat anti-mouse IgG (1:5000 dilution; KPL) was added to each well. After incubation for 60 min at 37°C, the plates were washed five times for 5 min with PBST. Finally, immune complexes were visualized by incubation with tetramethylbenzidine (TMB, Shanghai, Beyotime) for 10 min. The reaction was stopped by adding 2 M H_2_SO_4_ and the absorbance values were read at 450 nm using an Sunrise-Basic ELISA reader (Tecan, Männedorf, Switzerland). All samples were assayed in triplicate.The results showed that the titers of mouse anti-rEnApiAP2 and -rEnApiAP2tr pAb were 1:25 600 and 1:102 400, respectively (**Additional file 4: Figure S2**).

### Preparation of mAb against rEnApiAP2tr

To prepare monoclonal antibody (mAb) against rEnApiAP2tr, the spleen cells collected from the immunized mice were fused with SP2/0 myeloma cells using the previously described method (Kunert and Reinhart, 2016). Culture supernatants were screened by ELISA two weeks after fusion. Selected clones were subcloned using limiting dilutions. Hybridoma clones were isotyped using a commercial isotyping kit (Biodragon, Suzhou, China) by ELISA. Seven days after injection with about 1×10^6^ hybridoma cells, ascites fluids were withdrawn from pristane-primed BALB/c. The mAbs have been affinity purified using Protein G affinity chromatography medium kit (Genscript, Nanjing, China) according to manufacturer’s directions. The subtype identification of mAbs was carried out according to the manufacturer’s instructions (Biodragon, Suzhou, China). The level of mAb was assessed by ELISA method as described above.

### Western blot analysis

In order to identify recombinant proteins, the purified rEnApiAP2 and rEnApiAP2tr were separated by 6% and 12% SDS-PAGE and transferred to the nitrocellulose membrane (Merck Millipore, Billerica, MA, USA). The membranes were blocked with 3% BSA (Merck) in PBS overnight at 4°C. Subsequently, the anti-6×His mouse mAb (dilution: 1:20 000, BBI, Shanghai, China), the anti-rEnApiAP2 mouse pAb (dilution, 1:50), the anti-rEnApiAP2tr mouse pAb (dilution, 1:50), the purified anti-rEnApiAP2tr mouse mAb (dilution, 1:50) or the convalescent serum (dilution, 1:200) of chicken infected with *E. necatrix* were used as the primary antibodies and incubated at 37°C for 1 h, followed by three washes with 0.03% Tween-20/TBS (TBST) for 10 min each. HRP-conjugated goat anti-mouse IgG (dilution, 1:10 000; BBI, Shanghai, China) or goat anti-chicken IgG (dilution, 1:20 000, Jackson ImmunoResearch, Lancaster, U.S.A) were then used to detect the membrane-bound antibodies for 40 min at 37°C, respectively. The membranes were washed with TBST again for five times for 5 min and visualized with the manufacturer’s instructions of ECL ultrasensitive luminescence kit in a dark room. The membranes were exposed and photographed using the Tanon-5200 Chemiluminescent Imaging System. As a negative control, naive mouse and chicken sera were used.

### Detection of native EnApiAP2 protein

The soluble protein of SZ, MZ-2, MZ-3 and GAM were extracted from approximately 1×10^6^ SZ, MZ-2, MZ-3 or GAM according to the method described by Gao et al. (2021b). The nuclear protein of MZ-2, MZ-3 and GAM were extracted from approximately 1×10^6^ MZ-2, MZ-3 and GAM of *E. necatrix* using ProteinExt^®^ Mammalian Nuclear and Cytoplasmic Protein Extraction Kit (Transgen, Beijing, China) according to the manufacturer’s instructions, respectively. The protein concentration was determined using an Enhanced BCA Protein Assay Kit (Beyotime, Shanghai, China) according to the manufacturer’s instructions. Protein samples were separated by SDS-PAGE and then transferred to NC membrane for Western blot analysis as above. The anti-rEnApiAP2tr mouse mAb was used as the primary antibody (dilution, 1:200), and HRP-conjugated goat anti-mouse IgG (dilution, 1:10 000; BBI, Shanghai, China) was used as the secondary antibody. ECL staining was performed as above.

### Transcript levels of En*ApiAP2* in different developmental stages of *E. necatrix*

En*ApiAP2* cDNA was amplified from RNA extracted from SZ, MZ-2, MZ-3 and GAM of *E. necatrix*. A pair of specific primers (**Table 1**) was designed to amplify the EnApiAP2 cDNA by qPCR. The qPCR product was approximately 120 bp. The *E. necatrix* 5.8S ribosomal RNA coding sequence (AY943285) (**Table 1**) was used as an internal control. qPCR was performed using AceQ^®^ qPCR SYBR Green Master Mix (Vazyme, Nanjing, China) according to the manufacturer’s protocol. The reaction steps were: initial denaturation at 95°C for 5 min; denaturation at 95°C for 10 s, extension at 60°C for 30 s, amplification for 40 cycles. Each sample was carried out in biological triplicate. The relative expression of En*ApiAP2* mRNA was calculated through the 2^-ΔΔCt^ method (Livak and Schmittgen, 2001).

### Localization of EnApiAP2 in SZ, MZ-2 and MZ-3 of *E. necatrix*

Freshly purified SZ, MZ-2 and MZ-3 of *E. necatrix* were diluted in PBS and coated on glass slides, then fixed in pre-chilled methanol at −20°C for 15 min. After three washes PBST, the samples were permeabilized with 0.1% Triton^®^ X-100 at room temperature for 10 min and blocked with 3% (w/v) BSA in PBS at 37°C for 1.5 h. Then the slides were incubated with anti-rEnApiAP2tr mouse mAb (dilution, 1:200) or anti-rEnApiAP2 pAb (diluted 1:200) overnight at 4°C respectively, and incubated with fluorescein isothiocyanate (FITC)-conjugated goat anti-mouse IgG (dilution, 1:1 000; KPL, Maryland, U.S.A) for 1 h at 37°C. After incubated with DAPI-containing anti-fluorescence quenching tablets (Roche, Basel, Switzerland), the slides were analyzed by laser scanning confocal microscopy (LSCM) (Leica DM2500, Leica Microsystems GmbH, Wetzlar, Germany). The objective was 100× used to capture images for the immunofluorescence assay.

### Localization of EnApiAP2 expression in DF-1 cells

In order to detect the intracellular location of EnApiAP2 expression in DF-1 cells, the recombinant expression vectors of pEGFPC1-EnApiAP2 and pEGFPC1-ΔNLS-EnApiAP2 were constructed according to the method described previously (Wang et al., 2021). Briefly, coding sequence (CDS) of EnApiAP2 and ΔNLS-EnApiAP2 amplified using specific primers (**Table 1**) were cloned into pEGFP-C1 plasmid with *Bgl* II and *EcoR* I restriction sites using ClonExpress^®^ II One Step Cloning Kit (Vazyme, Nanjing, China) according to the manufacturer’s instructions, respectively. The vector construction strategy was shown in **Additional file 5 (Figure S3A)** and confirmed by PCR (**Additional file 5: Figure S3B**). Then, the pEGFPC1 and the recombinant plasmids pEGFPC1-EnApiAP2 and pEGFPC1-ΔNLS-EnApiAP2 were extracted using EndoFree Mini Plasmid Kit (TIANGEN, Beijing, China) according to the manufacturer’s instructions. After dilution with Opti-MEM, the plasmid DNA and Lipofectamin™ 3 000 (Thermo, Waltham, U.S.A) was mixed in a volume ratio of 1:1 and then transfected into chicken DF-1 cells. After incubation for 24 to 48 hours, the DF-1 cells were incubated with the anti-rEnApiAP2 mouse pAb (primary antibody) and then Cy3-conjugated goat anti-mouse antibody (second antibody). After incubated with DAPI-containing anti-fluorescence quenching tablets, the cells were analyzed by LSCM as above.

In addition, the nuclear and cytoplasmic proteins of DF-1 cells transfected with the recombinant plasmids were extracted and analyzed by Western blot as above, in which GFP mouse mAb (dilution, 1:10 00; Beyotime, Shanghai, China) was used as the primary antibody, HRP-conjugated goat anti-mouse IgG (dilution, 1:10 000; BBI, Shanghai, China) as the secondary antibody, histone H3 (dilution, 1:1000; Beyotime, Shanghai, China) as a cytosolic endogenous reference, and GAPDH (dilution, 1:1000; Beyotime, Shanghai, China) as a cytoplasmic endogenous reference, respectively. The localization of eukaryotic expressed EnApiAP2 was observed by the Tanon-5200 Chemiluminescent Imaging System. Furthermore, the soluble protein of the DF-1 cells transfected with the recombinant plasmids pEGFPC1-EnApiAP2 were extracted and analyzed by Western blot using the anti-rEnApiAP2 mouse pAb as above.

### Statistical analysis

All data were analyzed using GraphPad Prism software (version 8.0) and results are expressed as arithmetic mean ± standard deviation. At least three independent experiments were performed, and one-way analysis of variance (ANOVA) was used to analyze the data. Significant differences are indicated as *P < 0.05, **P < 0.01.

## Results

### Cloning and sequence analysis of EnApiAP2 gene

The cDNA sequence of EnApiAP2 from the YZ strain was amplified, resulting in a length of 5019 bp (**Additional file 3: Figure S1A**). This amplified sequence exhibited 100% identity to the sequence previously deposited in the NCBI database (XM_013579914.1). The amplified sequence encoded a polypeptide consisting of 1672 amino acids, with a predicted molecular mass of 176 kDa and an isoelectric point (pI) of 6.7, without a signal peptide (**Additional file 4: Figure S2A**) and transmembrane region (**Additional file 4: Figure S2B**), and with 61 antigenic epitopes (**Additional file 4: Figure S2C**). Sequence analysis revealed that EnApiAP2 contains an AP2 domain, which is composed of 52 amino acids (aa1101–1152) (**Figure 1A**) and contains 1 α-helix and 3 antiparallel β-strands in both the secondary structure (**Figure 1B**). In addition, EnApiAP2 also contains a ACDC (AP2-coincident C-terminal) domain and a NLS respectively (**Figure 1A**). The subcellular localization analysis revealed that EnApiAP2 protein was located in the nucleus (**Additional file 4: Figure S2D**). Homology of the full-length protein sequence of EnApiAP2 with *E. tenella* (XP_013233100.1), *E. praecox* (CDI87418.1), *E. acervulina* (XP_013247395.1), *E. brunetti* (CDJ50123.1), *Cyclospora cayetanensis* (XP_026194208.1), *Neospora caninum* (XP_003882378.1), *Toxoplasma gondii* (KFH05638.1), *Besnoitia besnoiti* (XP_029218535.1), *Cystoisospora suis* (PHJ16995.1) and *Plasmodium fragile* (XP_012335626.1) was 95.8% to 11.5%. Homology of the AP2 domain sequence of EnApiAP2 with above apicomplexan parasites was 100.0% to 36.5% (**Additional file 4: Figure S2E, S2F**), in which 14 amino acid residues were highly conserved (**Figure 1C**). The prediction of the three-dimensional structure showed that the AP2 domain of EnApiAP2 contains 1 alpha helix and 3 β fold (**Figure 1D**). According to software predictions, the full-length EnApiAP2 protein contains 64 phosphorylation sites, 15 propionylation sites, 8 sumoylation sites, 4 palmitoylation sites, 4 lysine acetylation sites, and 2 protein methylation sites. The phylogenetic analysis of AP2 domain sequences showed that EnApiAP2 with *E. tenella*, *E. praecox*, *E. acervulina*, *E. brunetti* and *C. cayetanensis* clustered in the same clade, whereas *N. caninum*, *T. gondii* and *B. besnoiti* clustered in another clade, and *P. fragile* formed a clade alone (**Figure 1E**).

### Expression, purification and identification of rEnApiAP2

**Figure 2:**
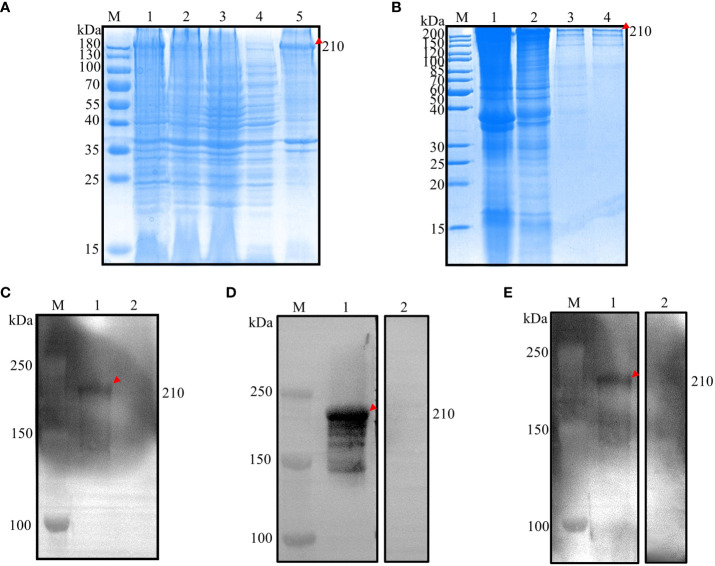
Prokaryotic expression analysis of EnApiAP2. **(A)** Inducible expression of rEnApiAP2. Lane 1: pET28a(+)-EnApiAP2a/BL21 induced by IPTG; Lane 2: pET28a(+)-EnApiAP2a/BL21 uninduced by IPTG; Lane 3: pET28a(+)/BL21 induced by IPTG; Lane 4: supernatant of bacterial sonicates; Lane 5: sediments of bacterial sonicates. **(B)** Purification of rEnApiAP2. Lane 1: lysate effluent after lysate binding to Ni-NTA; Lane 2: effluent of washing buffer; Lane 3: effluent of elution buffer; Lane 4: rEnApiAP2 after dialysis concentrate. **(C)** Expression of rEnApiAP2 detected by anti-His tag antibody. Lane 1: pET28a(+)-EnApiAP2a/BL21; Lane 2: pET28a(+)/BL21. **(D)** Reactivity analysis of rEnApiAP2. The primary antibody for lane 1 was chicken recovery serum anti-*E. necatrix* and for lane 2 was chicken-negative serum, respectively. **(E)** Immunogenicity analysis of rEnApiAP2. Lane 1: mouse anti-rEnApiAP2 pAb; Lane 2: mouse negative serum.

The construction of recombinant expression vectors pET28a(+)-EnApiAP2 was successfully accomplished, followed by their transformation into *E. coli* BL21(DE3) cells. These cells were subsequently induced with 0.6 mM IPTG for a duration of 4 hours at a temperature of 37°C. The obtained results indicated that the expressed recombinant protein (rEnApiAP2) had a molecular weight of approximately 210 kDa (as observed in **Figure 2A**, lane 1), which was slightly lower than the anticipated 181.4 kDa. Conversely, no protein was detected in the negative control (**Figure 2A**, lane 2, 3). The recombinant protein was primarily expressed in inclusion bodies (**Figure 2A**, lane 5) and subsequently purified using a Ni-NTA chromatography column (**Figure 2B**, lane 4).

Western blot analysis was used to further identify the recombinant protein. The results showed that when bacterial lysates containing the recombinant protein or purified recombinant protein were probed with the anti-6×His-tag mAb, a band of the expected size, 210 kDa, was detected (**Figure 2C**, lane 1), the convalescent chicken sera (**Figure 2D**, lane 1) or the anti-rEnApiAP2 mouse pAb (**Figure 2E**, lane 1). No protein was detected in the negative control (**Figure 2C**, lane 2; **Figure 2D**, lane 2; **Figure 2E**, lane 2).

### Expression, purification and identification of rEnApiAP2tr

**Figure 3:**
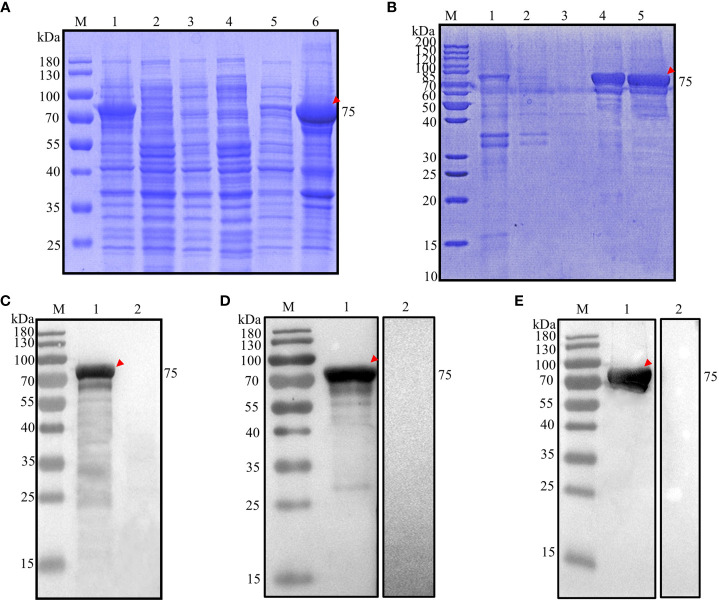
Analysis of prokaryotic expression of rEnApiAP2tr. **(A)** Inducible expression of rEnApiAP2tr. Lane 1: pET28a(+)-EnApiAP2tr/BL21 induced by IPTG; Lane 2: pET28a(+)-EnApiAP2tr/BL21 uninduced by IPTG; Lane 3: pET28a(+)/BL21 induced by IPTG; Lane 4: BL21 induced by IPTG; Lane 5: supernatant of bacterial sonicates; Lane 6: sediments of bacterial sonicates. **(B)** Purification of rEnApiAP2tr. Lane 1: lysate effluent after lysate binding to Ni-NTA; Lane 2: First washing buffer effluent; Lane 3: Third washing buffer effluent; Lane 4: effluent of elution buffer; Lane 5: rEnApiAP2tr after dialysis concentrate. **(C)** Expression of rEnApiAP2tr detected by anti-His tag antibody. Lane 1: pET28a(+)-EnApiAP2tr/BL21; Lane 2: pET28a(+)/BL21. **(D)** Reactivity analysis of rEnApiAP2. The primary antibody for lane 1 was chicken recovery serum anti-*E. necatrix* and for lane 2 was chicken-negative serum, respectively. **(E)** Immunogenicity analysis of rEnApiAP2tr. Lane 1: mouse anti-rEnApiAP2tr pAb; Lane 2: mouse negative serum.

A PCR product of approximately 1908 base pairs, encoding amino acid residues 1038-1672 (**Additional file 3: Figure S1B**), was inserted into a pET28a(+) expression vector containing a 6×His tag at the N-terminus. This construct was then introduced into chemically competent *E. coli* BL21 cells. The successfully transformed *E. coli* BL21 cells were subjected to induction with 1.0 mM IPTG at 37°C for 4 hours. The analysis revealed that the expressed recombinant protein had a molecular weight of approximately 75 kDa (**Figure 3A**, lane 1) and was predominantly found in inclusion bodies (**Figure 3A**, lane 6). No protein was detected in the negative control (**Figure 3A**, lanes 2-4). The recombinant protein was purified using the aforementioned method (**Figure 3B**, lane 5), and were precipitated with 8000. The final concentration of rEnApiAP2tr was 15 mg·L^-1^.

The recombinant protein was further identified by Werstern blot analysis as above. The result showed that a specific 75 kDa band was detected by the anti-6×His tag mAb (**Figure 3C**, lane 1), the convalescent chicken sera of *E. necatrix* (**Figure 3D**, lane 1) and the anti-rEnApiAP2tr mouse pAb (**Figure 3E**, lane 1). Negative control had no detection of the protein (**Figure 2C**, lane 2; **Figure 2D**, lane 2; **Figure 2E**, lane 2).

### Characterization of mAb against rEnApiAP2tr

**Figure 4:**
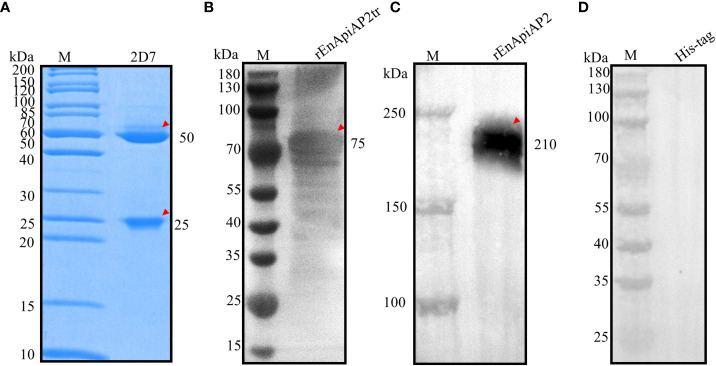
Preparation and characterization of EnApiAP2 mAb. **(A)** SDS-PAGE analysis of the purification of mAb ascites. Lane 1: 2D7. **(B)** Western blot analysis of rEnApiAP2tr by mAb 2D7. **(C)** Western blot analysis of rEnApiAP2 by mAb 2D7. **(D)** Western blot analysis of His-tag protein by mAb 2D7.

Following fusion, screening by ELISA and subcloned for three times by limiting dilutions, a hybridoma producing antibodies against rEnApiAP2tr were developed. The resulting mAb, designated mAb-2D7, reacted specifically with rEnApiAP2tr but not with His-tagged protein in Western blot assay (**Figure 4D**). The ascites of mice was purified by Protein G affinity chromatography (Genscript, Nanjing, China). One heavy chain with a size of 50 kDa and one light chain with a size of 25 kDa were observed in the purified monoclonal antibodies, and no other bands were observed, indicating that the purification efficacy of monoclonal antibodies was better (**Figure 4A**). The McAb-2D7 was identified as IgG1. The titer of purified mAb-2D7 was 1:819 200 (**Additional file 2: Table 2**). Western blot analysis showed that rEnApiAP2tr and rEnApiAP2 could be specifically recognized by mAb-2D7 (**Figures 4B, C**).

### The native EnApiAP2 and its transcript level in the different stages of *E. necatrix*

**Figure 5:**
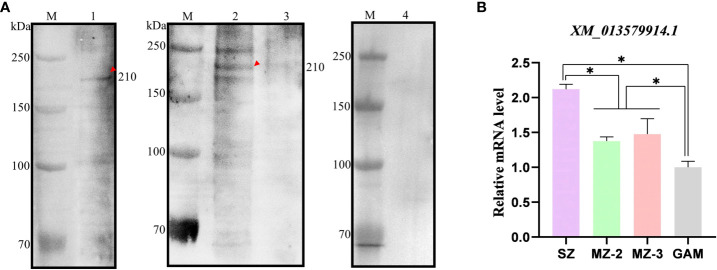
Recognition of native protein EnApiAP2 by mAb 2D7 and transcription level analysis of ENH_00027130. **(A)** Western blot analysis of native *Eimeria* proteins by mAb 2D7. Lane 1: native SZ protein; Lane 2: native MZ-2 nuclear protein; Lane 3: native MZ-3 nuclear protein; Lane 4: native GAM nuclear protein. **(B)** Transcription levels of EnApiAP2 in different developmental stages of *E. necatrix*. Significant differences are indicated as *P < 0.05.

The native EnApiAP2 was detected by use of Western blot analysis (**Figure 5A**). The results showed that a ~210 kDa band was specifically recognized by mAb-2D7 in the soluble protein of SZ, and in the nuclear protein of MZ-2 but not MZ-3 and GAM, which migrated slower than that expected based on the predicted size of 176 kDa. In addition, another two protein bands appeared at ~250 kDa and ~180 kDa in MZ-2 (**Figure 5A**).

The transcript levels of EnApiAP2 at different developmental stages of *E. necatrix* were detected by qPCR (**Figure 5B**). The transcript levels of EnApiAP2 in SZ were higher than that in MZ-2, MZ-3 and GAM (*P* < 0.05). The transcript levels of EnApiAP2 in MZ-2 and MZ-3 were higher than that in GAM (*P* < 0.05), but no significant difference between MZ-2 and MZ-3(*P* > 0.05).

### Localization of EnApiAP2 in the different stages of *E. necatrix* (*in vivo*) and DF-1 cells (*in vitro*)

**Figure 6:**
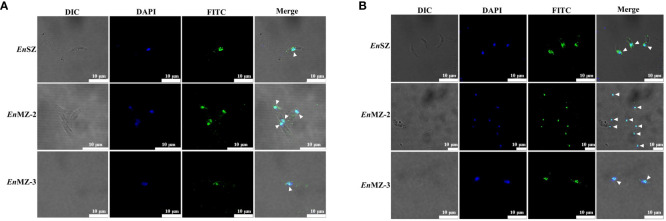
Localization of native EnApiAP2 protein at different endogenous development stages of *E. necatrix* by indirect immunofluorescence assay. **(A)** Mouse anti-rEnApiAP2 pAbs were used as primary antibodies, and nuclei were labeled with DAPI. **(B)** MAb 2D7 ascites were used as primary antibodies, and nuclei were labeled with DAPI. Scale bar=10 μm.

Localization of EnApiAP2 in SZ, MZ-2 and MZ-3 of *E. necatrix* was determined by IFA using anti-rEnApiAP2 mouse pAb and mAb-2D7 as primary antibody respectively, and FITC-conjugate goat anti-mouse IgG as secondary antibody. The results showed that obvious green fluorescence was observed in the nucleus of SZ, MZ-2 and MZ-3, while almost no green fluorescence was found in the cytoplasm, indicating that the EnApiAP2 protein was mainly localized in the nucleus of *E. necatrix* (**Figures 6A, B**).

**Figure 7:**
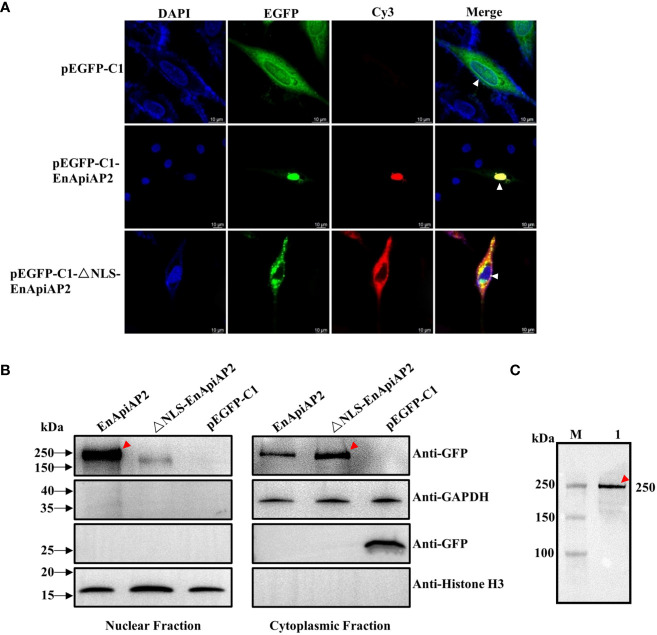
The NLS characterization of EnApiAP2. **(A)** LSCM analysis was performed to examine the localization of pEGFPC1-EnApiAP2 and pEGFPC1-ΔNLS-EnApiAP2 in DF-1 cells, with nuclei labeled with DAPI. Scale bar=10 μm. **(B)** Western-blot analysis was conducted to determine the distribution of pEGFPC1-EnApiAP2 and pEGFPC1-ΔNLS-EnApiAP2 protein in the nuclei or cytoplasm of DF-1 cells. **(C)** Western blot analysis of EnApiAP2 in DF-1 cells by mouse anti-rEnApiAP2 pAbs.

The pEGFPC1-EnApiAP2 and pEGFPC1-ΔNLS-EnApiAP2 fusion proteins were generated to investigate their intracellular localizations when overexpressed in DF1 cells. The findings revealed that the EnApiAP2 protein was predominantly localized in the nucleus of DF-1 cells, while the ΔNLS-EnApiAP2 protein was primarily localized in the cytoplasm (**Figure 7A**). To further confirm these localizations, the cytoplasmic and nuclear fractions were isolated and subjected to Western blot analysis. The results demonstrated the presence of EnApiAP2 in both the nuclear and cytoplasmic fractions, whereas ΔNLS-EnApiAP2 was exclusively detected in the cytoplasm fraction (**Figure 7B**). Furthermore, a ~250 kDa band in the soluble protein of the DF-1 cells transfected with pEGFPC1-EnApiAP2 was specifically recognized by the anti-rEnApiAP2 mouse pAb (**Figure 7C**). These results suggest that EnApiAP2 can be transferred to the nucleus via NLS in DF1 cells, and further imply that EnApiAP2 is a transcription factor.

## Discussion

AP2 (*Apetala2*) domains were initially characterized in the AP2/ERF protein family of plants (Jofuku et al., 1994), which represents the second largest group of transcription factors in *Arapidopsis thaliana* (Riechmann and Meyerowitz, 1998). These AP2/ERF proteins in plants serve as transcriptional activators or repressors (Riechmann and Meyerowitz, 1998) and consist of one or two AP2 DNA-binding domains, each comprising 60 amino acids. These domains bind DNA through a triple-stranded β-sheet, which is stabilized by a C-terminal α-helix (Allen et al., 1998). Comparative analysis of multiple AP2 domains has been conducted across various plant species and other organisms showed that 12 amino acid residues including V146, G148, V149, Y151, W158, A160, W162, W172, F176, A179, R180 and A183 were highly conserved (Balaji et al., 2005). ApiAP2 proteins were first identified in the apicomplexan genera *Plasmodium*, *Theileria* and *Cryptosporidium* (Balaji et al., 2005) and have subsequently been identified in all Apicomplexan genomes analyzed to date (Reid et al., 2014; Jeninga et al., 2019). Similar to plants, the ApiAP2s have a length of approximately 60 amino acids and can be found as single or tandem domains (Balaji et al., 2005). Although AP2 domain in ApiAP2 proteins is weakly homologous to the plant AP2 domain, the protein fold has been maintained between plants and the apicomplexan parasites, which normally consist of three β-strands and one C-terminal α-helix that stabilises the β-strands (Balaji et al., 2005; De Silva et al., 2008). The X-ray crystal structure of the DNA-bound *P. falciparum* AP2 domain PF14_0633 shows that a β-sheet fold binds the DNA major groove through base-specific and backbone contacts, and a prominent α-helix supports the β-sheet structure; the four key residues (N72, R74, R88, and S90) within the β-strand region directly contact the DNA (Lindner et al., 2010). These four amino acids are highly conserved in all of the Apicomplexan orthologs of PF14_0633, suggesting that the specificity of the DNA sequence is well conserved (De Silva et al., 2008; Lindner et al., 2010). In the present study, we cloned an *ENH_00027130* sequence from cDNA isolated from MZ-2 of *E. necatrix*, encoding 1672-aa protein. The protein deduced from the gene contains a 52 amino acids (aa1101-1152) AP2 domain, which consists of three antiparallel β-strands and one α-helix. In addition, of 12 highly conserved amino acid residues reported previously (Balaji et al., 2005), the eight also existed in the AP2 domain of EnApiAP2, including G5, V6, W15, A17, W19, F38, A4l and A45. The alignment of the AP2 domain revealed that the AP2 domain was highly conserved from EnApiAP2 (*ENH_00027130*) to orthologues in four additional avian *Eimeria* spp. and six Apicomplexan species.

The size of full-length ApiAP2 proteins exhibits significant variation, ranging from approximately 200 to several thousand amino acids. These proteins can possess one to four AP2 domains, along with supplementary functional regions. For instance, certain ApiAP2 proteins found in Plasmodium spp. feature a DNA binding domain known as AT-hook, a zinc finger domain, an Acyl-CoA-N-acetyltransferase domain, as well as a pentapeptide-repeat-like domain. Additionally, several *P. falciparum* ApiAP2 proteins contain an ACDC domain, primarily located at the C-terminus, referred to as the AP2-coincident domain (Jeninga et al., 2019; Chen et al., 2023). Furthermore, it is noteworthy that ApiAP2 proteins exhibit minimal homology beyond the AP2 domain, and even the paralogous AP2 domains found in *Plasmodium* spp. demonstrate limited sequence similarity among themselves (Jeninga et al., 2019). In this study, the sequence analysis revealed that the EnApiAP2 protein contains an ACDC domain apart from an AP2 domain. The AP2 domain was fundamentally conserved across six Eimeriidae species, four cyst-forming coccidia and *P. fragile*. The similarity between amino acid sequences of the full-length ApiAP2 protein was obviously lower than that of AP2 domains, except for *E. tenella* (95.8% and 100%, respectively). The phylogenetic analysis of AP2 domain sequences showed that six Eimeriidae species, four cyst-forming coccidia and *P. fragile* clustered in different clades, which was consistent with the previous research (Morrison et al., 2004).

In Western blot analysis with McAb-2D7, we found that the native EnApiAP2 protein was detected only in the whole-cell protein of SZ and in the nuclear protein of MZ-2. When analyzing transcript levels using qPCR, we found that EnApiAP2 transcript levels were significantly higher in SZ than in MZ-2, MZ-3 and GAM, and significantly higher in MZ-2 and MZ-3 than in GAM. These findings implied that EnApiAP2 might play a regulatory role on asexual rather than sexual replication. Interestingly, the size of the native EnApiAP2 protein was ~210 kDa in SZ and MZ-2, which was larger than the deduced theoretical molecular mass (~176 kDa). Furthermore, the size of the pEGFPC1-EnApiAP2 fusion protein expressed in DF-1 cells was ~250 kDa, which was also larger than expected from the theoretical molecular weights (~205 kDa). The reason for this observation was likely due to some post-translational modifications of EnApiAP2 protein as reported by Lee et al. (2023). In addition, another two protein bands in the nuclear protein of MZ-2 appeared at ~250 kDa and ~180 kDa, which may also imply that EnApiAP2 protein occur different degrees of modifications in the nuclear of MZ-2. Moreover, the molecular size of rEnApiAP2 prepared from bacteria (210 kDa) was larger than the predicted 181.4 kDa, which may be due to the denaturation and refolding of rEnApiAP2 proteins. The recombinant proteins cannot refold correctly under high concentration urea denaturation conditions, resulting in changes in molecular weight size (Singh and Panda, 2005).

Nuclear localization signal (NLS), a short amino acid sequence derived from eukaryotic nuclear proteins and viral proteins, has been found to efficiently mediate intranucleus transport of cargo molecules (Sun et al., 2016). The first identified NLS (PKKKRKV) from SV40 large T-antigen (Kalderon et al., 1984) has led to the discovery of numerous proteins containing NLS in the AP2 family of plants (Zhai et al., 2013; Djemal and Khoudi, 2015; Vatansever et al., 2017; Hu et al., 2020). NLS peptides can be classified into two categories: classical and non-classical sequences, as described by Deveshwar et al. (2020). Classical NLS peptides are characterized by the presence of either a single cluster of basic amino acid residues (monopartite) or two clusters of basic residues separated by 10-12 neutral residues (bipartite). In this study, ^1615^PLKKRTACARP^1625^ was predicted as a monopartite NLS of EnApiAP2. In the DF-1 cells, ΔNLS-EnApiAP2 could not enter the nucleus. This suggests that EnApiAP2 has the ability to penetrate the parasite’s cell nucleus through the NLS. Furthermore, IFA showed that EnApiAP2 was located in the nucleus of SZ, MZ-2 and MZ-3 of *E. necatrix*. Taken together, ApiAP2 protein encoded by *ENH_00027130* was a nuclear protein and might play a role as nuclear transcription factor. The known and *de novo* motifs to the promoters of target genes of EnApiAP2 were predicted by FIMO software (Version 5.5.4), and the follow-up EMSA test is in progress.

## Conclusion

In summary, an AP2 domain-containing protein (EnApiAP2) encoded by *ENH_00027130* sequence was successfully cloned and expressed in *E. coli* BL21(DE3). The AP2 domain is composed of 52 amino acids, in which 14 residues show a strong conservation, and consists of three antiparallel β-strands and one α-helix. EnApiAP2 protein was localized in the nucleus of *E. necatrix* parasites, and relied on the NLS for migration to DF-1 cell nucleus. Taken together, ApiAP2 protein might be a nuclear transcription factor. These results provide a basis for understanding of the role of EnApiAP2 protein regulating *E. necatrix* development.

## Data availability statement

The raw data supporting the conclusions of this article will be made available by the authors, without undue reservation.

## Ethics statement

This study was approved by the Animal Ethics Committee of Yangzhou University. All chickens and mice were handled in accordance with good animal practices required by the Animal Ethics Procedures and Guidelines of the People’s Republic of China. The study was conducted in accordance with the local legislation and institutional requirements.

## Author contributions

WC: Data curation, Investigation, Methodology, Validation, Writing – original draft, Writing – review & editing. QF: Investigation, Methodology, Writing – review & editing. LW: Investigation, Methodology, Writing – review & editing. SS: Data curation, Writing – review & editing. ZH: Data curation, Writing – review & editing. DL: Project administration, Writing – review & editing. XK: Writing – review & editing. JX: Data curation, Writing – review & editing. ZP: Writing – review & editing. JT: Funding acquisition, Project administration, Supervision, Writing – original draft, Writing – review & editing.

## Supplementary material

Supplementary Table 1 Indirect ELISA to determine optimal antigen encapsulation and serum dilution.

Supplementary Table 2 The potency and subclasses of purified mAb ascites.

Supplementary Figure 1 Cloning of full-length and truncated En*ApiAP2*. **(A)** Electrophoresis of En*ApiAP2* amplification products. **(B)** Electrophoresis of EnApiAP2tr amplification products.

Supplementary Figure 2 Different online websites predict information about EnApiAP2 protein. **(A)** signal peptide; **(B)** transmembrane region; **(C)** antigen determinant; **(D)** subcellular localization. **(E)** Comparison of amino acid sequences of full-length ApiAP2 in several species of Apicomplexa; **(F)** Comparison of amino acid sequences of AP2 domain of ApiAP2 in several species of Apicomplexa. 1: *E. necatrix* (XP_013435368.1), 2: *E. tenella* (XP_013233100.1), 3: *E. praecox* (CDI87418.1), 4: *E. acervulina* (XP_013247395.1), 5: *E. brunetti* (CDJ50123.1), 6: *C. cayetanensis* (XP_026194208.1), 7: *N. caninum* (XP_003882378.1), 8: *T. gondii* (KFH05638.1), 9: *B. besnoiti* (XP_029218535.1), 10: *C. suis* (PHJ16995.1), 11: *P. fragile* (CDI87418.1).

Supplementary Figure 3 ELISA titer of mouse pAb anti recombinant protein and Western blot analysis of native EnApiAP2. **(A)** rEnApiAP2; **(B)** rEnApiAP2tr; **(C)** Western blot analysis of EnApiAP2 in soluble protein of *E. necatrix.* 1: MZ-2, 2: MZ-3, 3: GAM.

Supplementary Figure 4 Analysis of the NLS of EnApiAP2. (A) The diagram of pEGFPC1-EnApiAP2 and pEGFPC1-ΔNLS-EnApiAP2; (B) The construction of pEGFPC1-EnApiAP2 and pEGFPC1-ΔNLS-EnApiAP2; M: GeneRuler 1Kb/DL 5000 Marker; 1: full-length EnApiAP2; 2: upstream fragment of △NLS-EnApiAP2; 3: downstream fragment of △NLS-EnApiAP2; 4: pEGFPC1-EnApiAP2; 5: pEGFPC1-△NLS-EnApiAP2.
