# Supplementary material for: Localization in vivo and in vitro confirms EnApiAP2 protein encoded by ENH_00027130 as a nuclear protein in Eimeria necatrix
Source: Front Cell Infect Microbiol. 2023 Dec 5;13:1305727. doi: 10.3389/fcimb.2023.1305727 (PMC10728482; doi:10.3389/fcimb.2023.1305727)
Supplement: Supplementary Table 1 — Indirect ELISA to determine optimal antigen encapsulation and serum dilution. [file Table_1.docx]

| Serum dilution | Test item | antigen concentration/μg·mL^-1^ | | | | | | |
| --- | --- | --- | --- | --- | --- | --- | --- | --- |
|  |  | 8 | 4 | 2 | 1 | 0.5 | 0.25 | 0.125 |
| 1:800 | P | 2.858 | 2.733 | 2.382 | 1.876 | 1.462 | 1.168 | 0.864 |
|  | N | 0.047 | 0.035 | 0.038 | 0.035 | 0.034 | 0.04 | 0.041 |
|  | P/N | 60.809 | 78.086 | 62.684 | 53.6 | 43 | 29.2 | 21.073 |
| 1:1600 | P | 2.841 | 2.742 | 2.208 | 1.832 | 1.445 | 1.132 | 0.751 |
|  | N | 0.043 | 0.038 | 0.038 | 0.036 | 0.036 | 0.039 | 0.036 |
|  | P/N | 66.07 | 72.158 | 58.105 | 50.889 | 40.139 | 29.026 | 20.861 |
| 1:3200 | P | 2.788 | 2.48 | 2.192 | 1.705 | 1.292 | 0.92 | 0.739 |
|  | N | 0.045 | 0.036 | 0.043 | 0.039 | 0.038 | 0.042 | 0.043 |
|  | P/N | 61.956 | 68.889 | 50.977 | 43.718 | 34 | 21.905 | 17.186 |
| 1:6400 | P | 2.358 | 2.222 | 2.16 | 1.658 | 1.281 | 0.87 | 0.722 |
|  | N | 0.039 | 0.033 | 0.036 | 0.033 | 0.034 | 0.038 | 0.037 |
|  | P/N | 60.462 | 67.333 | 60 | 50.242 | 37.676 | 22.895 | 19.514 |
| 1:12800 | P | 1.735 | 1.603 | 1.444 | 1.393 | 1.085 | 0.435 | 0.421 |
|  | N | 0.041 | 0.034 | 0.04 | 0.036 | 0.036 | 0.046 | 0.047 |
|  | P/N | 42.317 | 47.147 | 36.1 | 38.694 | 30.139 | 9.457 | 8.957 |
| 1:25600 | P | 1.121 | 0.978 | 0.904 | 0.685 | 0.475 | 0.287 | 0.206 |
|  | N | 0.038 | 0.034 | 0.039 | 0.034 | 0.034 | 0.044 | 0.037 |
|  | P/N | 29.5 | 28.765 | 23.179 | 20.147 | 13.971 | 6.523 | 5.568 |
| 1:51200 | P | 0.607 | 0.495 | 0.431 | 0.277 | 0.206 | 0.15 | 0.112 |
|  | N | 0.044 | 0.034 | 0.036 | 0.034 | 0.034 | 0.037 | 0.042 |
|  | P/N | 13.795 | 14.559 | 11.972 | 8.147 | 6.059 | 4.054 | 2.667 |
| 1:102400 | P | 0.263 | 0.224 | 0.194 | 0.153 | 0.104 | 0.086 | 0.07 |
|  | N | 0.038 | 0.034 | 0.038 | 0.034 | 0.047 | 0.043 | 0.039 |
|  | P/N | 6.921 | 6.588 | 5.105 | 4.5 | 2.213 | 2 | 1.795 |

**Table 1 Determination of optimal antigen encapsulation and optimal serum dilution by indirect ELISA**
