# Supplementary material for: Localization in vivo and in vitro confirms EnApiAP2 protein encoded by ENH_00027130 as a nuclear protein in Eimeria necatrix
Source: Front Cell Infect Microbiol. 2023 Dec 5;13:1305727. doi: 10.3389/fcimb.2023.1305727 (PMC10728482; doi:10.3389/fcimb.2023.1305727)
Supplement: Supplementary Table 2 — The potency and subclasses of purified mAb ascites. [file Table_2.docx]

| Hybridoma cell line | Cell supernatant titer | Unpurified ascites titer | Purified ascites titer | Antibody subclass |
| --- | --- | --- | --- | --- |
| 2D7 | 1∶12 800 | 1∶6 553 600 | 1∶819 200 | IgG1 |

**Table 2 The potency and subclasses of purified mAb ascites**
