# Supplementary figures and images for: Localization in vivo and in vitro confirms EnApiAP2 protein encoded by ENH_00027130 as a nuclear protein in Eimeria necatrix
Source: Front Cell Infect Microbiol. 2023 Dec 5;13:1305727. doi: 10.3389/fcimb.2023.1305727 (PMC10728482; doi:10.3389/fcimb.2023.1305727)

## Slide 1
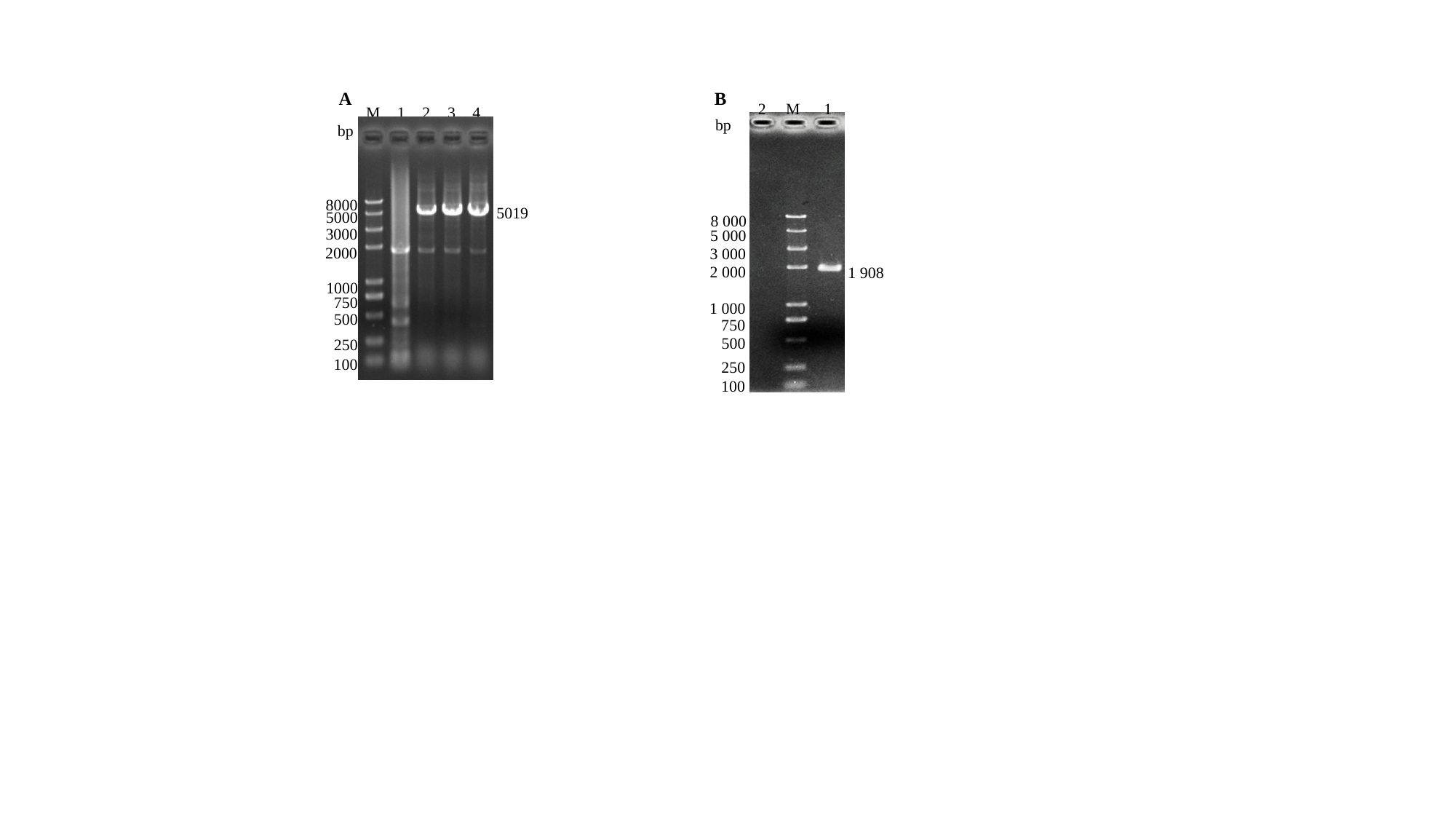

2 M 1
8 000
5 000
3 000
2 000
1 000
750
500
250
100
1 908
B
bp
A
M 1 2 3 4
bp
8000
5019 bp
5000
3000
2000
750
500
250
100
1000

Supplement: Supplementary Figure 1 — Cloning of full-length and truncated EnApiAP2. (A) Electrophoresis of EnApiAP2 amplification products. (B) Electrophoresis of EnApiAP2tr amplification products. [file Presentation_1.pptx]

## Slide 1
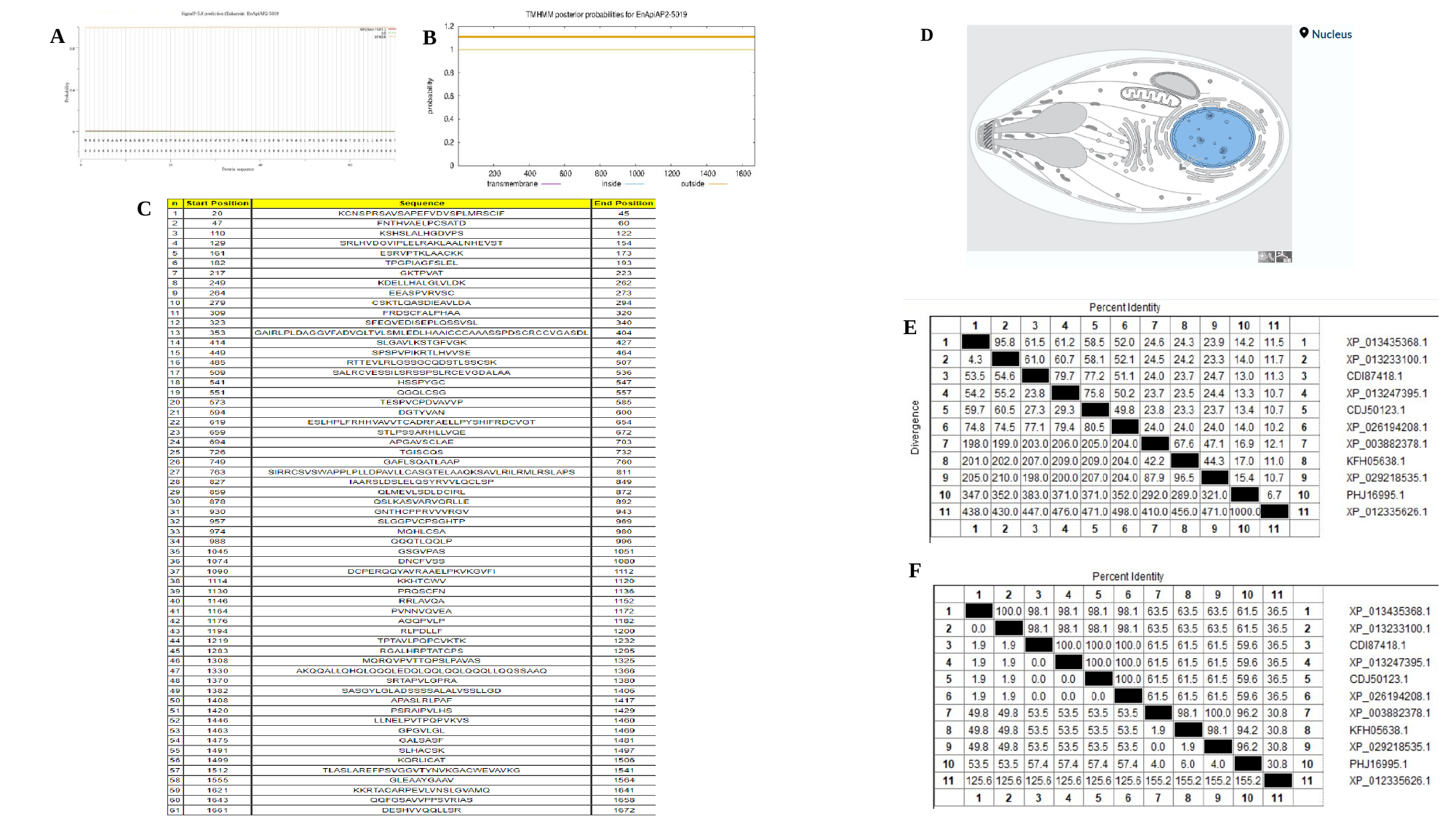

A
B
C
D
E
F

Supplement: Supplementary Figure 2 — Different online websites predict information about EnApiAP2 protein. (A) signal peptide; (B) transmembrane region; (C) antigen determinant; (D) subcellular localization. (E) Comparison of amino acid sequences of full-length ApiAP2 in several species of Apicomplexa; (F) Comparison of amino acid sequences of AP2 domain of ApiAP2 in several species of Apicomplexa. 1: E. necatrix (XP_013435368.1), 2: E. tenella (XP_013233100.1), 3: E. praecox (CDI87418.1), 4: E. acervulina (XP_013247395.1), 5: E. brunetti (CDJ50123.1), 6: C. cayetanensis (XP_026194208.1), 7: N. caninum (XP_003882378.1), 8: T. gondii (KFH05638.1), 9: B. besnoiti (XP_029218535.1), 10: C. suis (PHJ16995.1), 11: P. fragile (CDI87418.1). [file Presentation_2.pptx]

## Slide 1
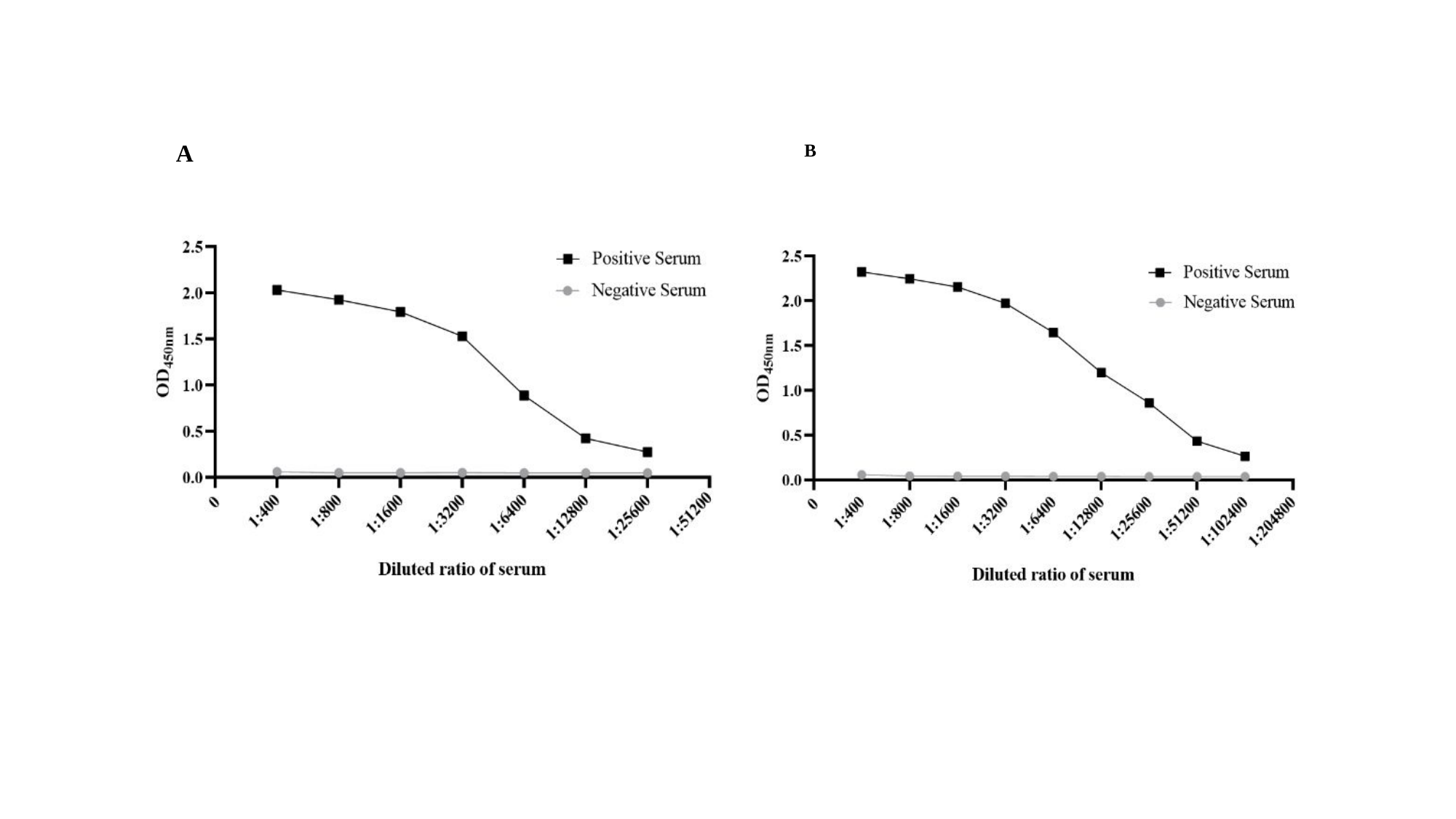

B
A

Supplement: Supplementary Figure 3 — ELISA titer of mouse pAb anti recombinant protein and Western blot analysis of native EnApiAP2. (A) rEnApiAP2; (B) rEnApiAP2tr; (C) Western blot analysis of EnApiAP2 in soluble protein of E. necatrix. 1: MZ-2, 2: MZ-3, 3: GAM. [file Presentation_3.pptx]

## Slide 1
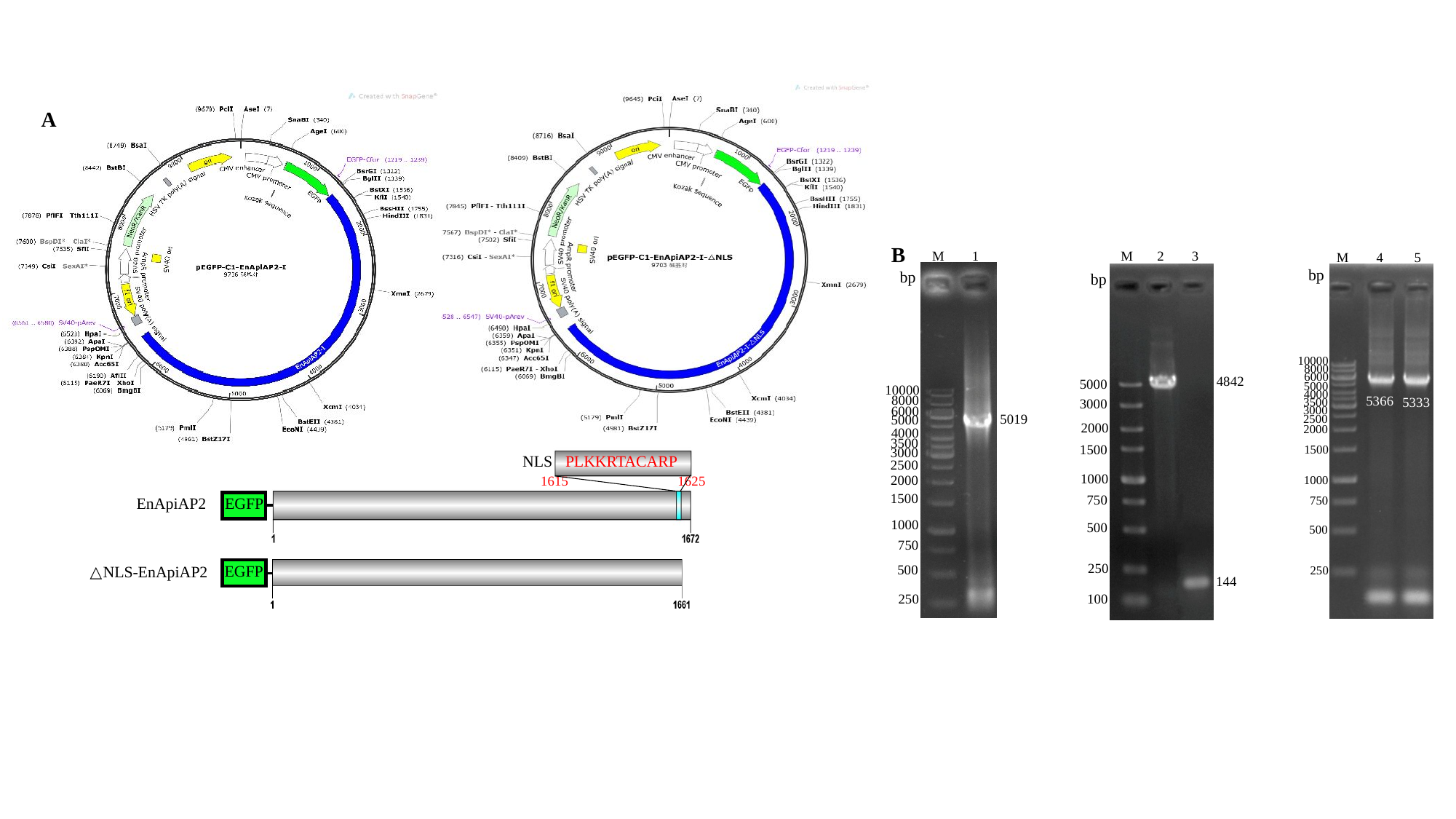

NLS
PLKKRTACARP
1615
1625
EGFP
EnApiAP2
EGFP
△NLS-EnApiAP2
A
B
M 1
10000
8000
6000
5000
4000
3500
2500
2000
1500
1000
750
500
250
3000
5019
bp
M 4 5
10000
8000
6000
5000
4000
3500
3000
2000
1500
1000
750
500
250
2500
5366
5333
bp
M 2 3
4842
5000
3000
2000
1500
1000
750
500
250
100
144
bp

Supplement: Supplementary Figure 4 — Analysis of the NLS of EnApiAP2. (A) The diagram of pEGFPC1-EnApiAP2 and pEGFPC1-ΔNLS-EnApiAP2; (B) The construction of pEGFPC1-EnApiAP2 and pEGFPC1-ΔNLS-EnApiAP2; M: GeneRuler 1Kb/DL 5000 Marker; 1: full-length EnApiAP2; 2: upstream fragment of △NLS-EnApiAP2; 3: downstream fragment of △NLS-EnApiAP2; 4: pEGFPC1-EnApiAP2; 5: pEGFPC1-△NLS-EnApiAP2. [file Presentation_4.pptx]
